# Efficient Total Synthesis of Lissodendrin B, 2-Aminoimidazole Marine Alkaloids Isolated from *Lissodendoryx* (*Acanthodoryx*) *fibrosa*

**DOI:** 10.3390/md18010036

**Published:** 2019-12-31

**Authors:** Xianfeng Wei, Xuelong Hu, Rilei Yu, Shengbiao Wan, Tao Jiang

**Affiliations:** 1 Key Laboratory of Marine Drugs, Chinese Ministry of Education, School of Medicine and Pharmacy, Ocean University of China, Yushan Road 5, Qingdao 266003, China; weixfeng427@163.com (X.W.); ryu@ouc.edu.cn (R.Y.); biaowan@ouc.edu.cn (S.W.); 2 Laboratory for Marine Drugs and Bioproducts, Qingdao National Laboratory for Marine Science and Technology, Qingdao 266003, China

**Keywords:** total synthesis, Lissodendrin B, 2-aminoimidazole, marine alkaloids

## Abstract

Lissodendrin B is a 2-aminoimidazole alkaloid bearing a (*p*-hydroxyphenyl) glyoxal moiety that was isolated from the Indonesian sponge *Lissodendoryx (Acanthodoryx) fibrosa.* We reported the first efficient total synthesis of Lissodendrin B. The precursor 4,5-disubstituted imidazole was obtained through Suzuki coupling and Sonogashira coupling reactions from 4-iodoimidazole. C2-azidation and reduction of the azide then provided the core structures of Lissodendrin B. Subsequent triple-bond oxidation, demethylation, and deacetylation gave the final product. The synthesis approach consists of ten steps with an overall yield of 1.1% under mild reaction conditions, and it can be applied for future analog synthesis and biological studies.

## 1. Introduction

Marine alkaloids offer significant advantages for the discovery of leading compounds because of their unique, complex structures and diverse bioactivities [1]. Unfortunately, most marine alkaloids are isolated in very small quantities, which limits further studies to generate combinatorial libraries for drug discovery and compound leads optimization. Therefore, efficient chemical synthesis of marine alkaloids in greater quantities is necessary for their usage in bioactivity studies. 2-aminoimidazole alkaloids, the most commonly investigated representative marine alkaloid, are found primarily in calcareous sponges, especially in the genera *Leucetta* and *Clathrina* [2,3,4,5]. These compounds have been extensively studied because of their various biological activities, including anticancer [3,4,5], antimicrobial [2,6], antivirus properties [7,8], P-gp-mediated MDR reversal activity [9] as well as leukotriene B4 receptor [10] and epidermal growth factor (EGF) receptor antagonist activities [11]. Therefore, efficient synthesis of 2-aminoimidazole compound is subject to increasing demand. There are two major approaches for preparation of 2-aminoimidazole compound: (1) the condensation of α-haloketone with an acetylated guanidine [12] or condensation of an α-aminoketone with cyanamide [13] and (2) functionalization of imidazole scaffold via protection, C2-amination, and deprotection [14,15].

**Figure 1:**
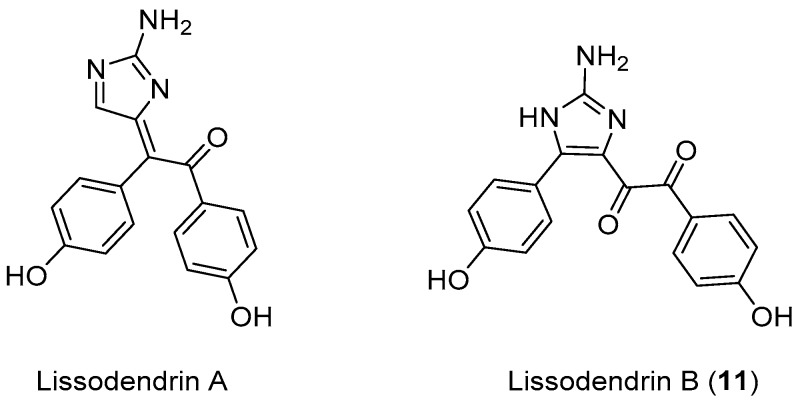
Structures of Lissodendrin A and Lissodendrin B.

The secondary metabolites, Lissodendrin A and Lissodendrin B (Figure 1) were isolated from the ethyl acetate fraction of the sponge *Lissodendoryx (Acanthodoryx) fibrosa* in 2016 and structural elucidation of these compounds was achieved using spectroscopic techniques. Lissodendrin A and Lissodendrin B possess unprecedented 2-aminoimidazole skeletons with the latter compound bearing a (*p*-hydroxyphenyl) glyoxal moiety, which is rarely encountered in natural products. The new natural alkaloid 11 is devoid of cytotoxicity to the L5178Y mouse lymphoma cell line at a dose of 10 μg/mL [16]. The precedent of diverse biological activity of 2-aminoimidazole alkaloids, coupled with the lack of synthetic methods available to date, argues well for its synthesis to support further biological evaluation. Herein, we reported the first successful and efficient total synthesis of Lissodendrin B involving Suzuki coupling and Sonogashira coupling reactions and we constructed the precursor 4,5-disubstituted imidazole. C2-azidation and reduction of the azide then provided the core structures of Lissodendrin B, and subsequent triple-bond oxidation, demethylation, and deacetylation led to the completion of the synthesis. All the compounds thus synthesized were fully characterized by ^1^H, ^13^C, and HRMS. 

## 2. Results and Discussion

### 2.1. Retrosynthetic Analysis of Lissodendrin B

**Scheme 1:**
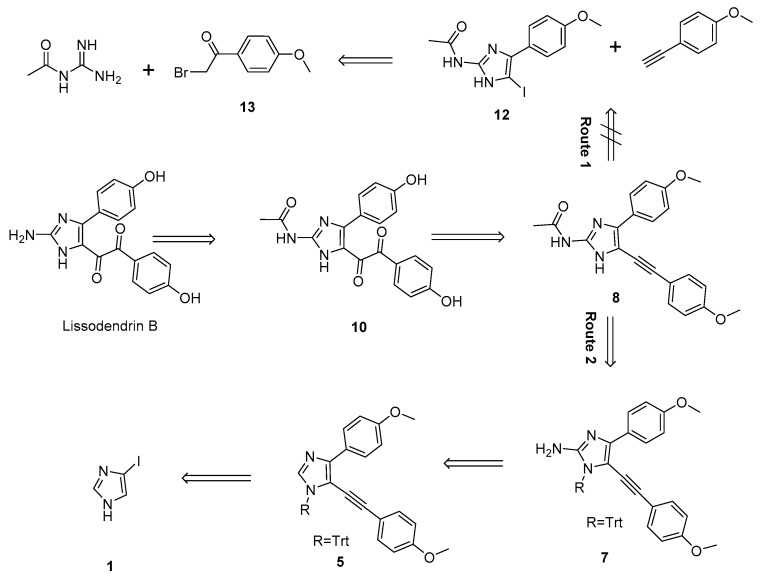
Retrosynthetic analysis of Lissodendrin B.

Scheme 1 illustrates the retrosynthetic pathway of Lissodendrin B. From the retrosynthetic analysis, we envisioned that the natural product could be produced by deacetylation [17] from acetamide **10**, which was obtained from triple-bond oxidation [18,19] and demethylation [20] of the key intermediate **8**. We initially anticipated that compound **8** could be produced through Sonogashira coupling reaction [21] from compound **12**, whose core moiety, 2-aminoimidazole skeletons, could be prepared by the condensation of α-haloketone **13** with an acetylated guanidine (Route 1) [12]. However, when we applied this strategy to our compound synthesis, the formation of compound **8** proceeded unsuccessfully via Sonogashira coupling reaction. We speculated that the electron-donating effect of acetamino group generated increasing electron clouds at the imidazole ring, leading to the reaction not occurring. Then, we attempted another approach (Route 2), in which the 2-amineimidazolone skeleton of intermediate **8** was constructed from the C2-azidation [14,15] and reduction [22] from intermediate **5**, which could be prepared by Sonogashira coupling reaction and Suzuki coupling reaction [23] from easily accessible starting material **1**.

### 2.2. Synthesis of Lissodendrin B

**Scheme 2:**
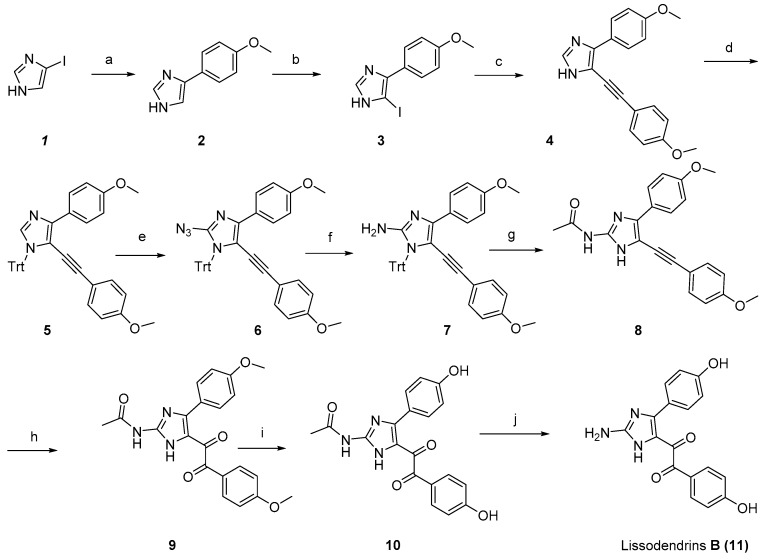
Synthesis of Lissodendrin B. Reagent and conditions: (**a**) 4-methoxyphenylboronic acid, Pd(PPh_3_)_4_, CsF, toluene, H_2_O, 100 °C, 24 h, 82% (**b**) NIS, CH_2_Cl_2_, r.t., 4 h, 79%; (**c**) 4-methoxyphenylacetylene, Pd(PPh_3_)_4_, CuI, TEA, DMF, 80 °C, 8 h, 70%; (**d**) Ph_3_CCl, TEA, CH_2_Cl_2_, reflux, 4 h, 77%; (**e**) n-C_4_H_9_Li, TsN_3_, THF, −78 °C, 6 h, 46%; (**f**) Na_2_S⸱9H_2_O, CH_3_OH; r.t., 4 h, 69%; (**g**) (1) Ac_2_O, TEA, CH_2_Cl_2_, reflux, 4 h; (2) HCl, CH_3_OH, r.t., 4 h, 62%; (**h**) Hg(NO_3_)_2_, DMF, r.t., 4 h, 51%; (**i**) BBr_3_, CH_2_Cl_2_, r.t., 4 h, 66%; (**j**) concentrated H_2_SO_4_, CH_3_OH, H_2_O, reflux, 4 h, 51%.

Scheme 2 shows a successful synthetic approach developed for production of Lissodendrin B. The synthesis commenced with the preparation of 4-(4-methoxyphenyl)-*1H*-imidazole **2**, using Pd(PPh_3_)_4_ as the catalyst and CsF as the base. The 4-iodoimidazole was treated with 4-methoxyphenyl boronic acid via Suzuki−Miyaura cross-coupling reaction in combined solution of toluene and water (V/V = 5/1) afforded the desired cross-coupling product **2** at a yield of 82% [23]. To introduce the 4-methoxyphenylacetylene by Sonogashira coupling reaction, iodization of arylimidazole **2** with NIS provided iodoimidazole **3** as a white solid at a yield of 79% [24]. The optimal conditions entailed the slow addition of 1.2 equiv. of NIS to avoid the formation of diiodide side-product. Sonogashira reactions of compound **3** with 4-methoxyphenylacetylene proceeded smoothly in the presence of Pd(PPh_3_)_4_ and CuI as catalysts and triethylamine as the base, affording coupling products **4** at a yield of 70% as a light-yellow liquid [21]. Using triethylamine as a catalyst, protection of the imidazole nitrogen with triphenylmethyl chloride at 45 °C in CH_2_Cl_2_ gave the known *N*-trityl imidazole **5** at 77% yield as a white solid [25]. Deprotonation of the imidazole **5** at C2 positions with n-BuLi in THF at −78 °C and trapping with TsN_3_ provided the azide **6** at 46% yield as a white solid [14,15]. Subsequent treatment with Na_2_S⸱9H_2_O in methanol at room temperature led to the reduction of the azide to amine **7** at good yield as a brown-yellow solid [22]. Acetylation of amine **7** with acetic anhydride in CH_2_Cl_2_ in the presence of triethylamine at reflux gave the corresponding acetamide, which was treated with concentrated hydrochloric acid causing removal of the trityl group, resulting in the expected compound **8** forming at a yield of 62% (in two steps).

With amide **8** in hand, oxidation of the triple-bond was performed to construct the corresponding α-diketone structure. At the outset, KMnO_4_ and NaHCO_3_ were used to convert compound **8** to α-diketone **9** [18]. The desired compound **9** was formed, but the yield thereof was poor (17%). Numerous attempts to optimize the reaction conditions through varying KMnO_4_ and NaHCO_3_ equivalents, solvent or order of addition were unsuccessful (Table 1). Subsequently, mercuric nitrate hydrate was used for this transformation [19]. It was encouraging that the triple-bond of amide **9** was successfully converted into α-diketone **9** at a yield of 51% as a yellow solid using mercuric nitrate hydrate (2 equiv.) in DMF at room temperature.

At this juncture, with core structure **9** in hand, we further performed functional modification, including demethylation and deacetylation: to our surprise, demethylation of α-diketone **9** was accomplished using BBr_3_ (5 equiv.) in CH_2_Cl_2_ at ambient temperature giving the corresponding diphenol **10** at a yield of 66% as a yellow solid [20]. Finally, the acetyl moiety of diphenol **10** was removed by using concentrated sulfuric acid in combined solution of methanol and water (V/V = 2/1) at 80 °C to give Lissodendrin B at a yield of 51% as a yellow solid [17]. It is noteworthy that the α-diketone moiety of Lissodendrin B is stable below 80 °C.

Thus, we completed the first total synthesis of Lissodendrin B in ten steps with an overall yield of 1.1%. Spectra of the synthesized Lissodendrin B were in excellent agreement with that of the natural product [16].

## 3. Materials and Methods

### 3.1. General Information

Dichloromethane and tetrahydrofuran were dried by distillation. All reagents used in the experiments were obtained from commercial sources without further purification. Reactions were monitored by thin layer chromatography (TLC). Visualization was achieved under a UV lamp (254 nm and 365 nm), and developed the plates with potassium permanganate. Flash column chromatography was performed on a silica gel (200–300 mesh). ^1^H NMR and ^13^C NMR spectra were taken on Jnm-Ecp-600 spectrometer, respectively, ^1^H NMR and ^13^C NMR spectra were referenced to tetramethylsilane (Me_4_Si). High resolution (ESI) MS spectra were recorded using a QTOF-2 Micromass spectrometer (Supplementary Materials).

### 3.2. Methods

#### 3.2.1. Synthesis of 4-(4-Methoxyphenyl)-1*H*-imidazole (**2**)

To a solution of 4-iodoimidazole **1** (15 g, 77.3 mmol ) in toluene (300 mL) and H_2_O (100 mL) was sequentially added 4-methoxyphenylboronic acid (23.5 g, 155 mmol), Pd(PPh_3_)_4_ (8.9 g, 7.7 mmol), CsF (23.5 g, 155 mmol) and N_2_ was bubbled it for 3–5 min. The heterogeneous mixture was stirred at 100 °C for 24 h under N_2_ atmosphere. The reaction mixture was cooled to room temperature and water was added, layers were separated. The aqueous layer was extracted with ethyl acetate (2 × 200 mL). The combined organic extracts were washed with brine, dried (anhydrous Na_2_SO_4_) and concentrated. The crude residue was purified by flash chromatography (CH_2_Cl_2_/CH_3_OH, 30:1) to give product **2** (11 g, 82%) as a white solid. ^1^H NMR (500 MHz, DMSO-*d*_6_) δ 12.13 (s, 1H), 7.66 (d, *J* = 8.9 Hz, 3H), 7.41 (s, 1H), 6.92 (d, *J* = 8.7 Hz, 2H), 3.75 (s, 3H). ^13^C NMR (101 MHz, DMSO-*d*_6_) δ 159.13, 138.80, 128.71, 114.41, 55.66. HRMS calcd for C_10_H_11_ON_2_ [M + H]^+^ 175.0866, found 175.0864. Spectroscopic data agrees with those previously reported [23].

#### 3.2.2. Synthesis of 5-Iodo-4-(4-methoxyphenyl)-1*H*-imidazole (**3**)

To a stirring solution of Compound **2** (11 g, 63.1 mmol) in anhydrous CH_2_Cl_2_ (300 mL) was added NIS (17.0 g, 75.7 mmol) in several portions. After stirring for 4 h at room temperature. The solvent was removed under vacuum, the residue was poured into saturated NaHCO_3_ solution and extracted with ethyl acetate (2 × 300 mL). The combined organic layers were washed with water brine, dried (anhydrous Na_2_SO_4_) and concentrated. The crude residue was purified by silica gel column chromatography (CH_2_Cl_2_/CH_3_OH, 50:1) to give compound **3** (15 g, 79%) as a white solid. ^1^H NMR (500 MHz, DMSO-*d*_6_) δ 12.65 (s, 1H), 7.62 (m, 3H), 7.03 (d, *J* = 8.2 Hz, 2H), 3.79 (s, 3H). ^13^C NMR (101 MHz, DMSO-*d*_6_) δ 158.30, 136.32, 136.06, 125.98, 114.43, 113.40, 55.52. HRMS calcd for C_10_H_10_ON_2_I [M+ H]^+^ 300.9832, found 300.9826.

#### 3.2.3. Synthesis of 5-(4-Methoxyphenyl)-4-((4-methoxyphenyl)ethynyl)-1*H*-imidazole (**4**)

To a solution of Compound **3** (15 g, 50 mmol) in DMF (200 mL) was added 4-methoxyphenylacetylene (9.9 g, 75 mmol), Pd(PPh_3_)_4_ (5.8 g, 5 mmol), CuI (950 mg, 5 mmol), triethylamine (208 mL, 150 mmol) and N_2_ was bubbled it for 3–5 min. The reaction mixture was stirred at 100 °C for 8 h under N_2_ atmosphere. The reaction mixture was cooled to room temperature and the solvent was removed under vacuum. The residue was poured into water and extracted with ethyl acetate (2 × 300 mL). The combined organic layers were washed with water, brine, dried (anhydrous Na_2_SO_4_) and concentrated. The crude residue was purified by silica gel column chromatography (CH_2_Cl_2_/CH_3_OH, 50:1) to give compound **4** (10.6 g, 70%) as a yellow oil. ^1^H NMR (400 MHz, Chloroform-d) δ 8.04 (d, *J* = 8.8 Hz, 2H), 7.66 (s, 1H), 7.44 (d, *J* = 8.8 Hz, 2H), 6.97 (d, *J* = 8.9 Hz, 2H), 6.87 (d, *J* = 8.8 Hz, 2H), 3.84 (s, 3H). 3.83 (s, 3H). ^13^C NMR (101 MHz, Chloroform-*d*) δ 159.77, 159.17, 135.03, 132.84, 127.34, 124.81, 115.08, 114.13, 114.08, 94.17, 80.25, 55.35, 55.32. HRMS calcd for C_19_H_17_O_2_N_2_ [M + H]^+^ 305.1285, found 305.1276.

#### 3.2.4. Synthesis of 5-(4-Methoxyphenyl)-4-((4-methoxyphenyl)ethynyl)-1-trityl-1*H*-imidazole (**5**)

To a stirring solution of Compound **4** (10 g, 32.9 mmol) in anhydrous CH_2_Cl_2_ (200 mL) was added triphenylmethyl chloride (13.8 g, 49.4 mmol) and trimethylamine (22.8 mL, 164.5 mmol). After stirring for 4 h at reflux, the solvent was removed under vacuum, the residue was poured into saturated NaHCO_3_ solution and extracted with ethyl acetate (2 × 200 mL). The combined organic layers were washed with water, brine, dried (anhydrous Na_2_SO_4_) and concentrated. The crude residue was purified by silica gel column chromatography (petroleum ether/ethyl acetate, 4:1) to give compound **5** (13.8 g, 77%) as a white solid. ^1^H NMR (400 MHz, Chloroform-*d*) δ 8.15 (d, *J* = 8.9 Hz, 2H), 7.48 (d, *J* = 0.9 Hz, 1H), 7.34–7.28 (m, 15H), 6.95 (d, *J* = 8.9 Hz, 2H), 6.78 (d, *J* = 8.8 Hz, 2H), 6.72 (d, *J* = 8.9 Hz, 2H), 3.85 (s, 3H), 3.78 (s, 3H). ^13^C NMR (101 MHz, Chloroform-*d*) δ 159.39, 159.07, 145.40, 141.73, 139.11, 132.23, 130.24, 128.16, 127.94, 127.81, 127.73, 127.55, 126.85, 115.22, 113.70, 112.98, 100.30, 80.45, 76.07, 55.33, 55.25. HRMS calcd for C_38_H_31_O_2_N_2_ [M + H]^+^ 547.2380, found 547.2378.

#### 3.2.5. Synthesis of 2-Azido-5-(4-methoxyphenyl)-4-((4-methoxyphenyl)ethynyl)-1-trityl-1*H*-imidazole (**6**)

Compound **5** (3.0 g, 5.5 mmol) in anhydrous THF (200 mL) was cooled to −78 °C and n-BuLi (11 mL, 27.5 mmol, 2.5 M in solution in THF) was added dropwise. After complete addition of the n-BuLi, the reaction was stirred for 2 h at −78 °C, then TsN_3_ (4.4 g, 22.0 mmol) dissolved in anhydrous THF (15 mL) was added. The reaction mixture was allowed to room temperature and stirred for 4 h. The reaction was quenched carefully with saturated aqueous NH_4_Cl and extracted with ethyl acetate (2 × 200 mL). The combined organic layers were washed with water, brine, dried (anhydrous Na_2_SO_4_) and concentrated. The residue was purified by silica gel column chromatography (petroleum ether/ethyl acetate, 15:1) to give compound **6** (1.5 g, 46%) as a white solid. ^1^H NMR (400 MHz, Chloroform-*d*) δ 8.10 (d, *J* = 8.8 Hz, 2H), 7.54–7.47 (m, 6H), 7.33–7.28 (m, 6H), 7.27–7.22 (m, 3H), 6.93 (d, *J* = 9.0 Hz, 2H), 6.83 (d, *J* = 9.0 Hz, 2H), 6.76 (d, *J* = 8.9 Hz, 2H), 3.85 (s, 3H), 3.81 (s, 3H).^13^C NMR (101 MHz, Chloroform-*d*) δ 159.33, 159.17, 145.33, 143.69, 142.28, 142.03, 134.67, 133.00, 132.40, 129.87, 129.74, 127.97, 127.87, 127.63, 127.22, 126.98, 126.25, 115.35, 113.73, 113.60, 111.11, 101.86, 81.30, 76.52, 55.32, 55.28. HRMS calcd for C_38_H_30_O_2_N_5_ [M + H]^+^ 588.2325, found 588.2328.

#### 3.2.6. Synthesis of 5-(4-Methoxyphenyl)-4-((4-methoxyphenyl)ethynyl)-1-trityl-1*H*-imidazol-2-amine (**7**)

To a stirring solution of Compound **6** (1.5 g, 2.6 mmol) in CH_3_OH (100 mL) was added Na_2_S⸱9H_2_O (5.3 g, 26 mmol). After stirring for 4 h at room temperature, the solvent was removed under vacuum. The residue was poured into water and extracted with ethyl acetate (2 × 100 mL). The combined organic layers were washed with water, brine, dried (anhydrous Na_2_SO_4_) and concentrated to give compound **7** (1 g, 69%) as a yellow solid. (Compound **7** was used in the next step without further purification.) ^1^H NMR (500 MHz, Chloroform-*d*) δ 8.00 (d, *J* = 8.5 Hz, 2H), 7.52 (m, 6H), 7.31 (m, 6H), 7.27 (dd, *J* = 7.4, 1.2 Hz, 3H), 6.87 (d, *J* = 8.5 Hz, 2H), 6.72 (m, 4H), 4.04 (s, 2H), 3.80 (s, 3H), 3.77 (s, 3H). ^13^C NMR (101 MHz, Chloroform-*d*) δ 158.98, 158.91, 149.86, 142.62, 142.43, 132.08, 130.34, 128.04, 127.81, 127.66, 126.97, 116.26, 113.76, 113.60, 108.06, 100.37, 83.02, 75.82, 55.41, 55.37. HRMS calcd for C_38_H_32_O_2_N_3_ [M + H]^+^ 562.2489, found 562.2481.

#### 3.2.7. Synthesis of *N*-(5-(4-Methoxyphenyl)-4-((4-methoxyphenyl)ethynyl)-1*H*-imidazol-2-yl) Acetamide (**8**)

To a stirring solution of Compound **7** (1.0 g, 1.8 mmol) in anhydrous CH_2_Cl_2_ (50 mL) was added triethylamine (2.5 mL, 18.0 mmol) and acetic anhydride (0.9 mL, 9.0 mmol). After stirring for 4 h at reflux, the solvent was removed under vacuum, the residue was dissolved in CH_3_OH (50 mL) and concentrated hydrochloric acid (5 mL) was added at 0 °C. After stirring for 4 h at room temperature, the solvent was removed under vacuum. The residue was poured into saturated NaHCO_3_ solution and extracted with ethyl acetate (2 × 100 mL). The combined organic layers were washed with water, brine, dried (anhydrous Na_2_SO_4_) and concentrated. The crude residue was purified by silica gel column chromatography (petroleum ether/ethyl acetate, 2:1) to give compound **8** (400 mg, 62%) as a pale yellow solid. ^1^H NMR (500 MHz, DMSO-*d*_6_) δ 11.88 (s, 1H), 11.31 (s, 1H), 7.98 (s, 2H), 7.48 (d, *J* = 8.2 Hz, 2H), 7.00 (m, 4H), 3.79 (s, 3H), 3.78 (s, 3H), 2.08 (s, 3H). ^13^C NMR (126 MHz, DMSO-*d*_6_) δ 169.09, 159.79, 158.83, 141.39, 140.22, 132.76, 129.12, 127.07, 126.94, 115.15, 114.94, 114.37, 103.97, 95.27, 80.60, 55.76, 55.57, 23.28. HRMS calcd for C_21_H_20_O_3_N_3_[M + H]^+^ 362.1499, found 362.1496.

#### 3.2.8. Synthesis of *N*-(5-(4-Methoxyphenyl)-4-(2-(4-methoxyphenyl)-2-oxoacetyl)-1*H*-imidazol-2-yl) Acetamide (**9**)

To a stirring solution of Compound **8** (360 mg, 1.0 mmol) in DMF (30 mL) was added Hg(NO_3_)_2_⸱1/2H_2_O (667.2 mg, 2.0 mmol). After stirring for 4 h at room temperature, the solvent was removed under vacuum. The residue was poured into water and extracted with ethyl acetate (2 × 50 mL). The combined organic layers were washed with water brine, dried (anhydrous Na_2_SO_4_) and concentrated. The crude residue was purified by silica gel column chromatography (petroleum ether/ethyl acetate, 2:1) to give compound **9** (200 mg, 51%) as a yellow solid. **[Caution: Mercury salts are highly toxic. Handling of all mercury compounds should be done carefully.]** ^1^H NMR (500 MHz, DMSO-*d*_6_) δ 12.50 (s, 1H), 11.21 (s, 1H), 7.92 (m, 2H), 7.80 (m, 2H), 7.07 (m, 4H), 3.84 (s, 3H), 3.83 (s, 3H), 2.01 (s, 3H). ^13^C NMR (101 MHz, DMSO-*d*_6_) δ 194.48, 190.76, 169.87, 164.56, 160.71, 145.96, 141.69, 136.84, 132.16, 130.89, 129.05, 126.31, 120.63, 115.01, 114.13, 56.20, 55.82, 23.15. HRMS calcd for C_21_H_20_O_5_N_3_[M + H]^+^ 394.1397, found 394.1392.

#### 3.2.9. Synthesis of *N*-(5-(4-Hydroxyphenyl)-4-(2-(4-hydroxyphenyl)-2-oxoacetyl)-1*H*-imidazol-2-yl) Acetamide (**10**)

To a stirring solution of Compound **9** (200 mg, 0.5 mmol) in anhydrous CH_2_Cl_2_ (25 mL) was added BBr_3_ (48 µL, 2.5 mmol) at 0 °C. The mixture was allowed to room temperature and stirred for 4 h. The mixture was neutralized carefully with saturated aqueous NaHCO_3_ at 0 °C and extracted with CH_2_Cl_2_ (2 × 50 mL). The combined organic layers were washed with water, brine, dried (anhydrous Na_2_SO_4_) and concentrated. The crude residue was purified by silica gel column chromatography (CH_2_Cl_2_/CH_3_OH, 30:1) to give compound **10** (120 mg, 66%) as a yellow solid. ^1^H NMR (500 MHz, DMSO-*d*_6_) δ 12.34 (s, 1H), 11.20 (s, 1H), 10.63 (s, 1H), 9.93 (s, 1H), 7.80 (d, *J* = 8.3 Hz, 2H), 7.68 (d, *J* = 8.5 Hz, 2H), 6.88 (t, *J* = 9.0 Hz, 4H), 2.01 (s, 3H). ^13^C NMR (125 MHz, DMSO-*d*_6_) δ 194.34, 190.93, 169.94, 163.59, 159.18, 142.52, 141.48, 132.43, 130.87, 128.77, 125.02, 119.08, 116.27, 115.48, 110.74, 107.32, 23.13. HRMS calcd for C_19_H_16_O_5_N_3_ [M + H]^+^ 366.1084, found 366.1076.

#### 3.2.10. Synthesis of Lissodendrin B (**11**) 

To a stirring solution of Compound **10** (100 mg, 0.27 mmol) in CH_3_OH (30 mL) and water (5 mL) was added concentrated H_2_SO_4_ (1 mL) at 0 °C. After stirring for 4 h at 80 °C, the reaction mixture was neutralized carefully with saturated aqueous NaHCO_3_ at 0 °C and extracted with ethyl acetate (2 × 50 mL). The combined organic layers were washed with water, brine, dried (anhydrous Na_2_SO_4_) and concentrated. The crude residue was purified by silica gel column chromatography (CH_2_Cl_2_/CH_3_OH, 30:1) to give Lissodendrin **B** (45 mg, 51%) as a yellow solid. ^1^H NMR (500 MHz, CH_3_OH-*d*_4_) δ 7.60 (d, *J* = 8.7 Hz, 2H), 7.14 (m, 2H), 6.78 (d, *J* = 8.7 Hz, 2H), 6.54 (d, *J* = 8.5 Hz, 2H). ^13^C NMR (125 MHz, CH_3_OH-*d*_4_) δ 194.32, 184.00, 164.90, 159.74, 155.32, 154.43,133.39, 132.07, 126.79, 124.69, 123.36, 116.47, 115.57. HRMS calcd for C_17_H_14_O_4_N_3_[M + H]^+^ 324.0979, found 324.0974.

## 4. Conclusions

In summary, a concise total synthesis of marine alkaloids Lissodendrin B was accomplished in ten steps giving an overall yield of 1.1%. Highlights of the synthesis included: (1) the precursor 4,5-disubstituted imidazole construction based on Suzuki coupling and Sonogashira coupling reactions, (2) 2-aminoimidazole skeleton synthesis using C2-azidation and reduction of the azide, and (3) the α-diketone structure preparation based on the oxidation of the triple-bond. Cost-effective reagents and mild reaction conditions were used in each step of our route. Results from this study are useful for design and synthesis of novel bioactive 2-aminoimidazole alkaloids. Further biological activity studies are underway and will be reported in due course.

## Figures and Tables

**Table 1:** Optimization of oxidation of the triple-bond.

Catalyst (equiv.)	Base	Solvent	Temperature	Time (h)	Yield (%)
KMnO_4_ (2 equiv.)	NaHCO_3_ (1 equiv.)	Acetone	0 °C -r.t.	4	17
KMnO_4_ (2 equiv.)	NaHCO_3_ (1 equiv.)	THF	0 °C -r.t.	8	10
KMnO_4_ (4 equiv.)	NaHCO_3_ (2 equiv.)	Acetone	0 °C -r.t.	8	20
Hg(NO_3_)_2_ (2 equiv.)	—	DMF	0 °C -r.t.	2	51

## Supplementary Materials

Supplementary materials can be found at https://www.mdpi.com/1660-3397/18/1/36/s1. Figures S1–S20: Copies of ^1^H and ^13^C NMR spectra of compounds **2**–**11**.

## Abbreviations

P-gp	P-glycoprotein
MDR	multidrug resistance
DMF	*N*,*N*-Dimethylformamide
TEA	Triethylamine
Ac_2_O	Acetic anhydride
DMSO	Dimethyl sulfoxide
THF	Tetrahydrofuran
TsN_3_	*p*-toluenesulfonyl azide
CsF	cesium fluoride
NIS	*N*-iodosuccinimide
CuI	copper(I) iodide
THF	Tetrahydrofuran
UV	ultraviolet
