# Supplementary material for: Efficient Total Synthesis of Lissodendrin B, 2-Aminoimidazole Marine Alkaloids Isolated from Lissodendoryx (Acanthodoryx) fibrosa
Source: Mar Drugs. 2019 Dec 31;18(1):36. doi: 10.3390/md18010036 (PMC7024156; doi:10.3390/md18010036)
Supplement: Supplementary file 1 [file marinedrugs-18-00036-s001.pdf]

Supplementary Material

Efficient total synthesis of Lissodendrin B, 2-aminoimidazole marine alkaloids isolated from *Lissodendoryx (Acanthodoryx) fibrosa*

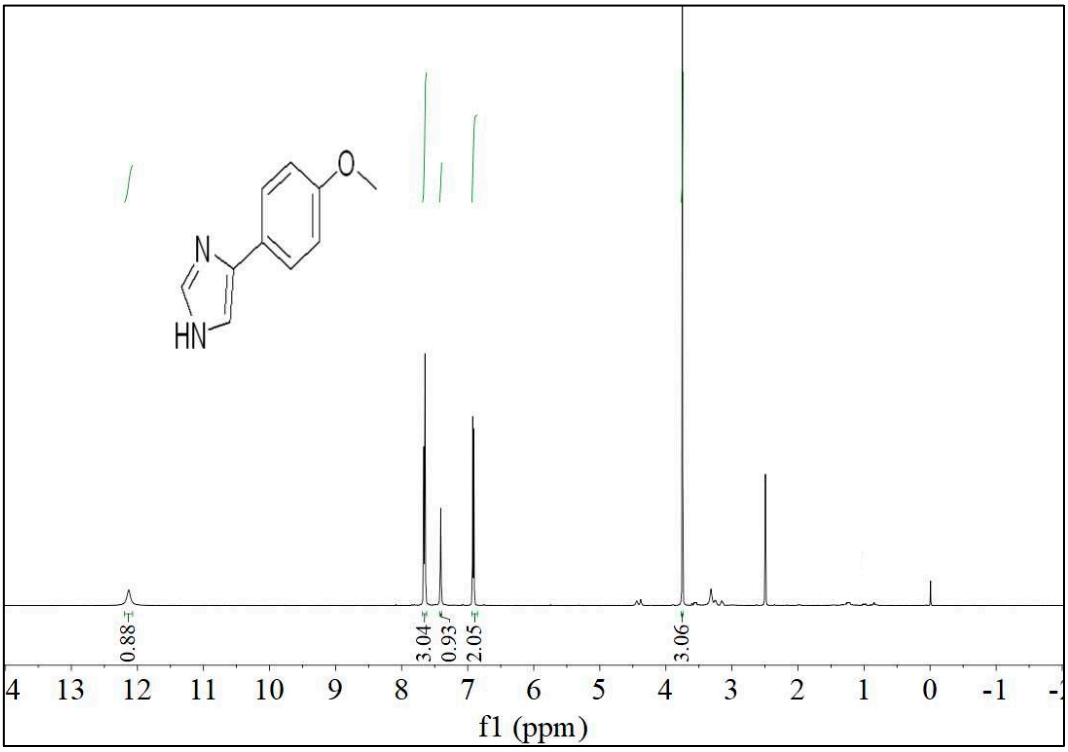

Figure S1 ^{1}H NMR Spectrum of **2** in DMSO-d$_6$

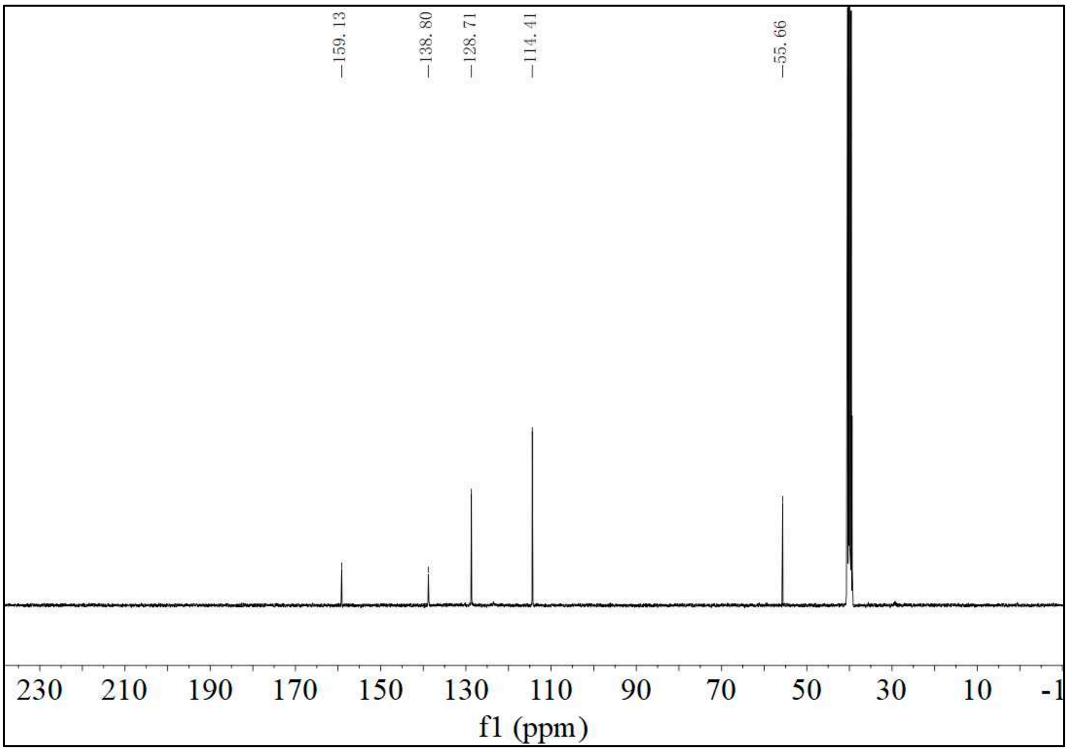

Figure S2 ^{13}C NMR Spectrum of **2** in DMSO-d$_6$

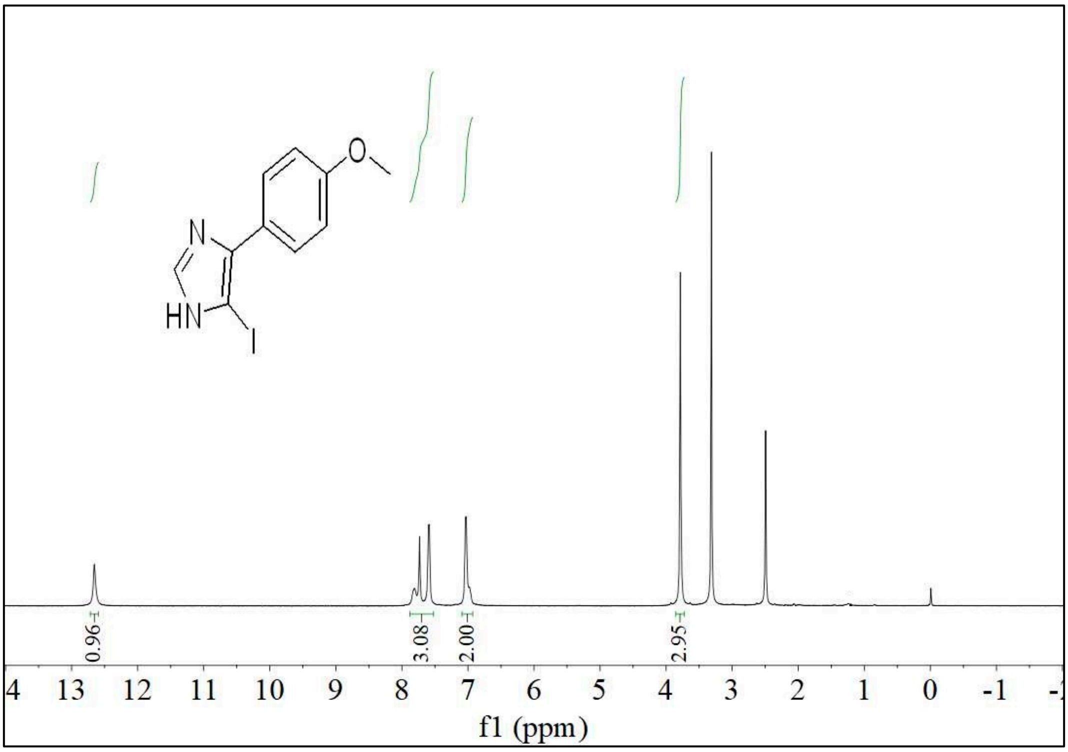

Figure S3 ^{1}H NMR Spectrum of **3** in DMSO-d$_6$

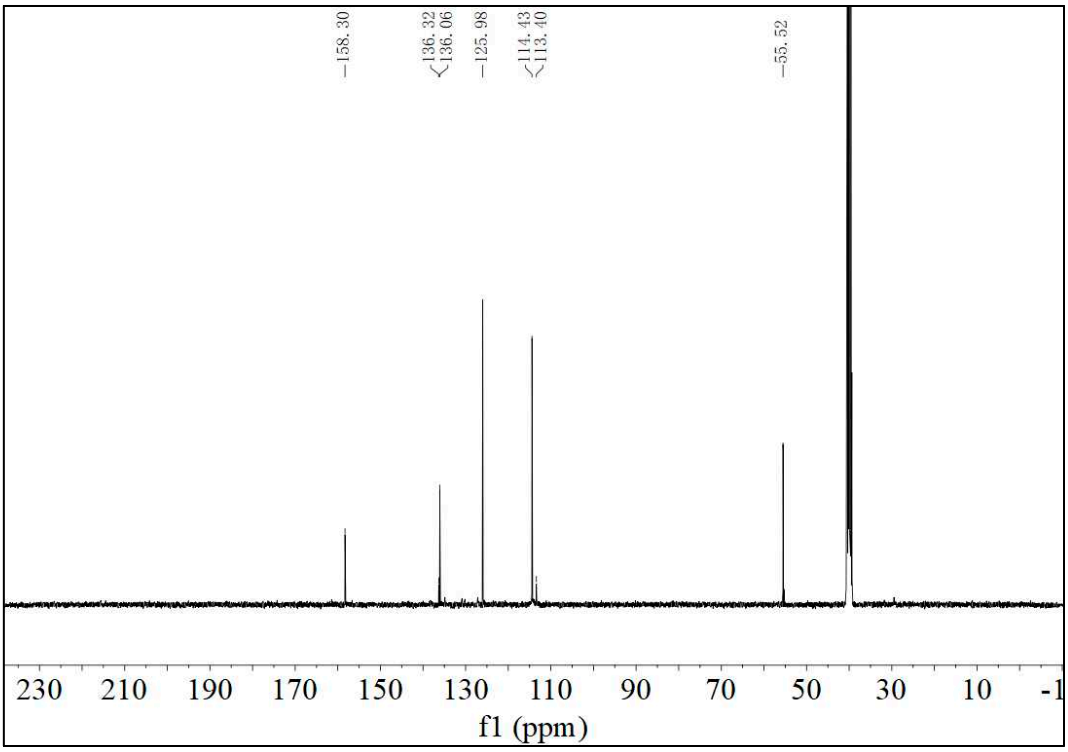

Figure S4 ^{13}C NMR Spectrum of **3** in DMSO-d$_6$

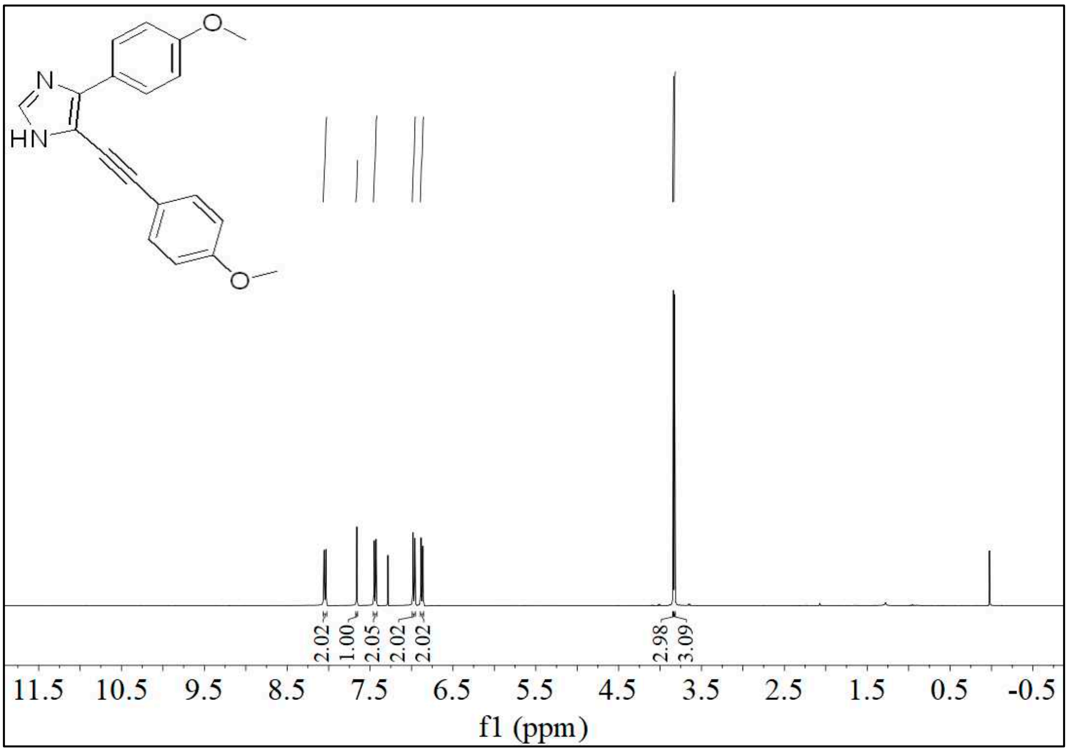

Figure S5 ¹H NMR Spectrum of **4** in Chloroform-d

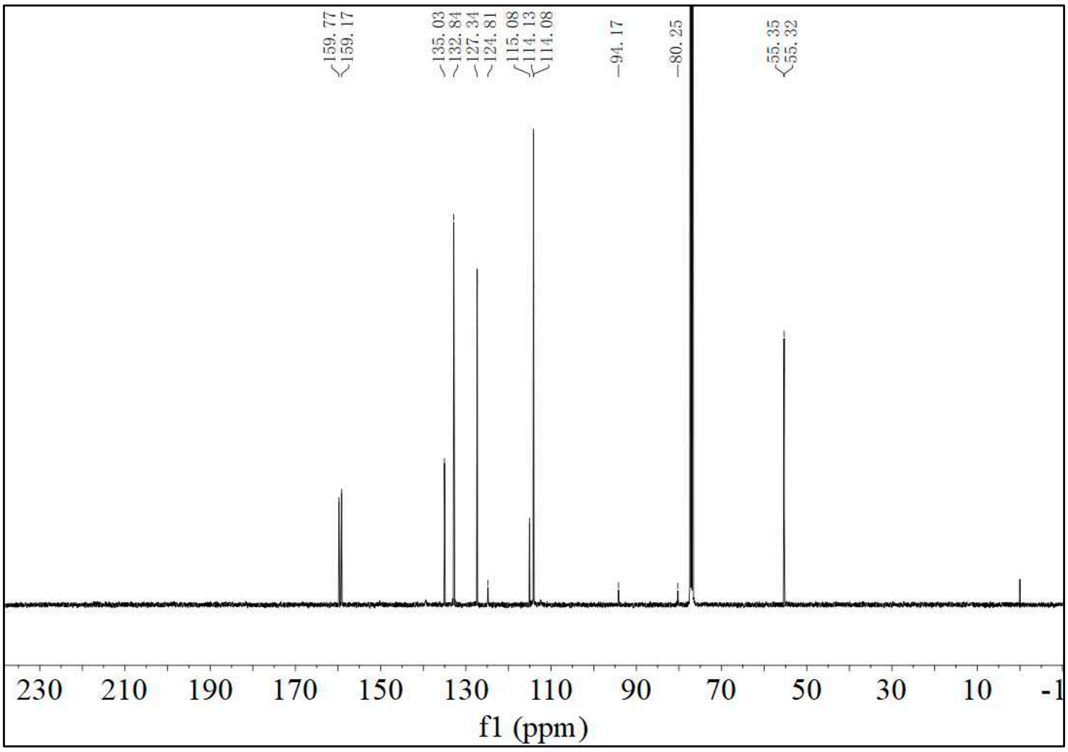

Figure S6 ¹³C NMR Spectrum of **4** in Chloroform-d

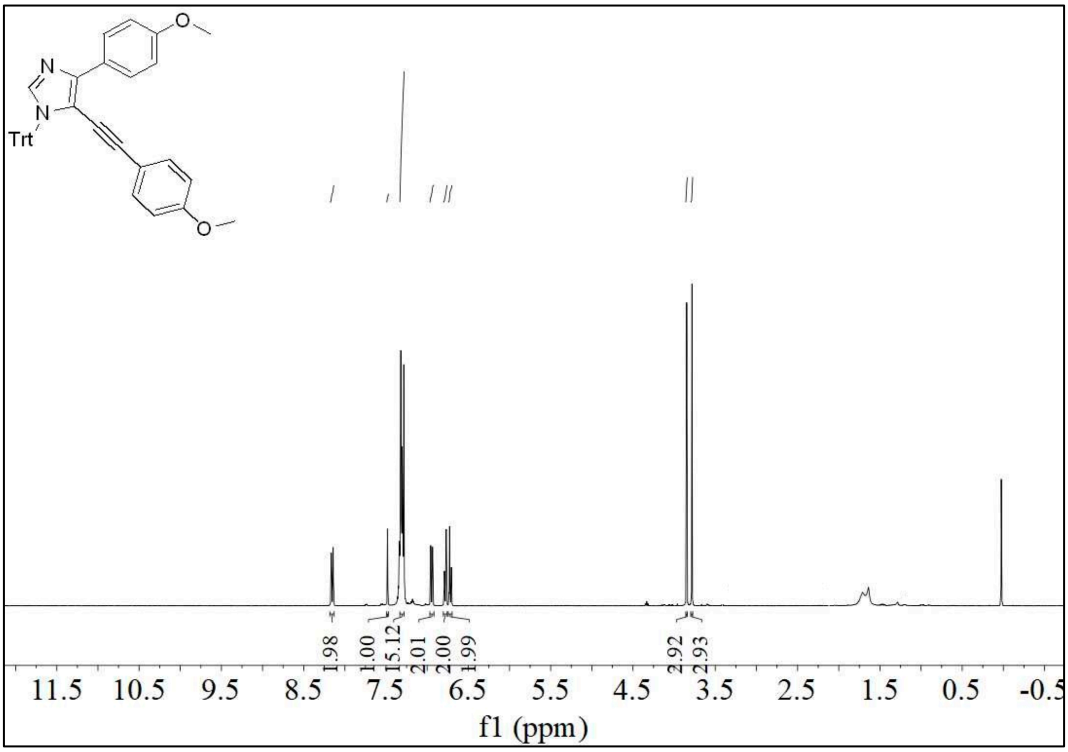

Figure S7 ^{1}H NMR Spectrum of **5** in Chloroform-d

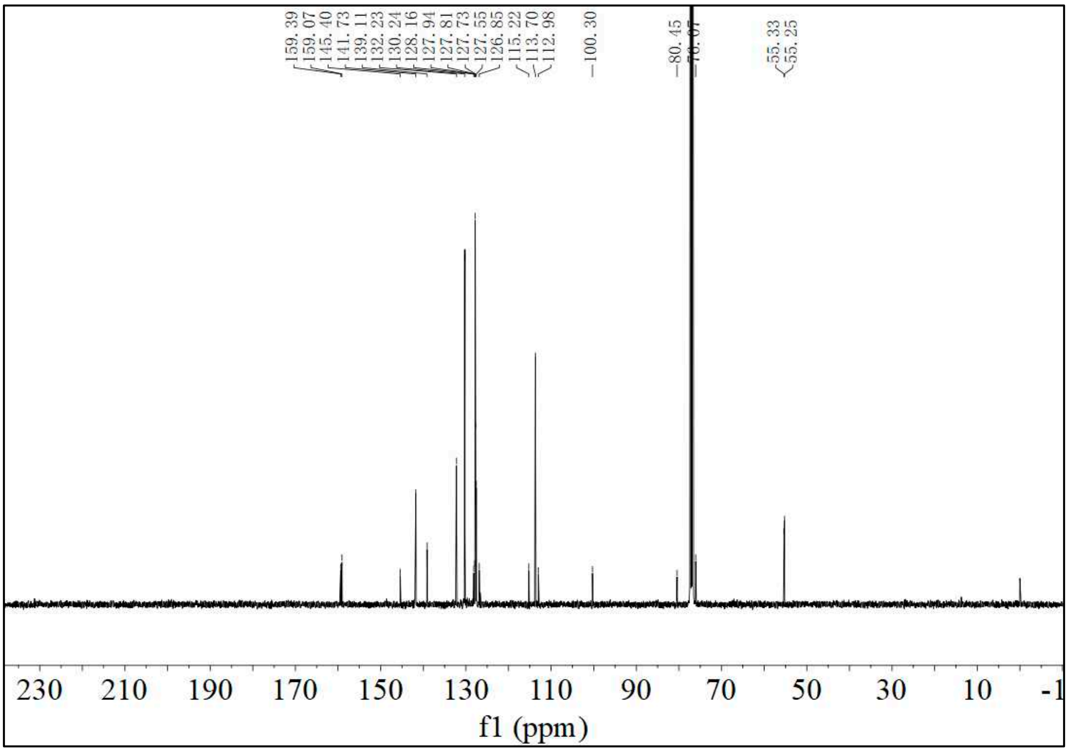

Figure S8 ^{13}C NMR Spectrum of **5** in Chloroform-d

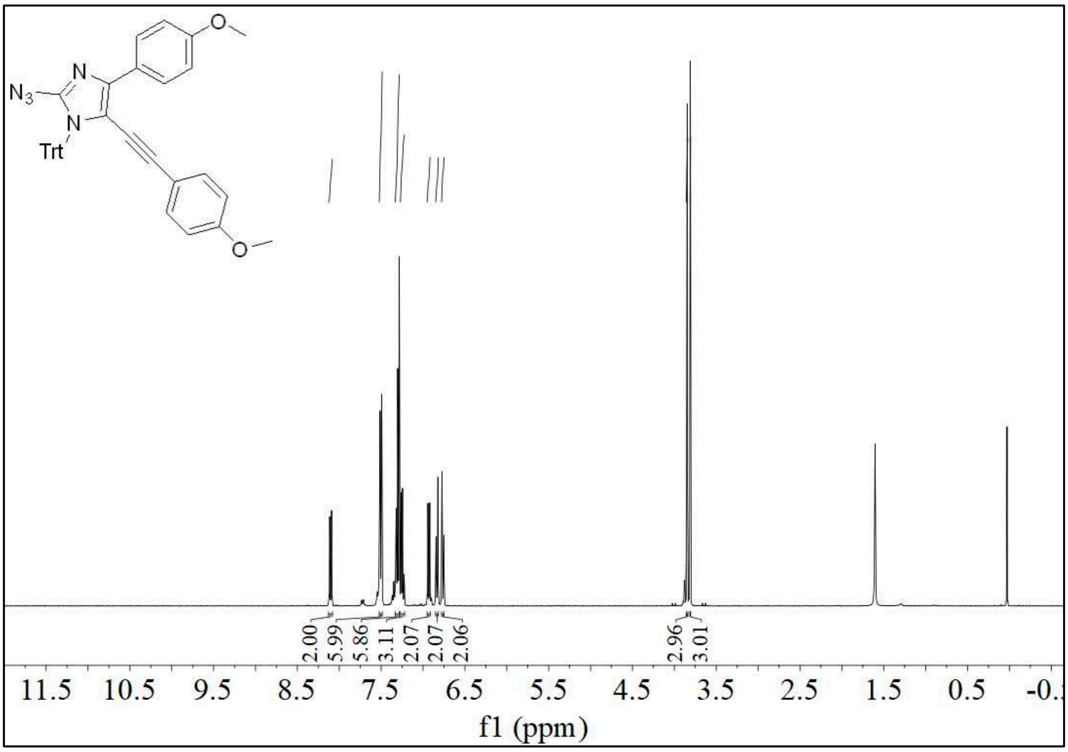

Figure S9 ^{1}H NMR Spectrum of **6** in Chloroform-d

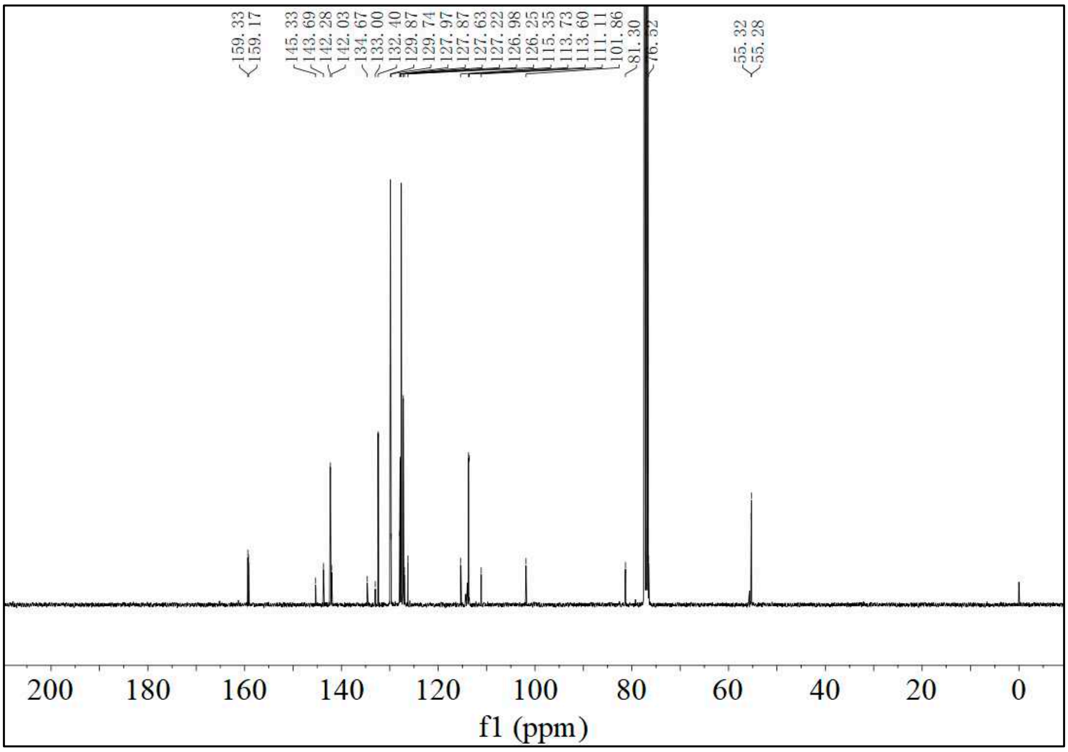

Figure S10 ^{13}C NMR Spectrum of **6** in Chloroform-d

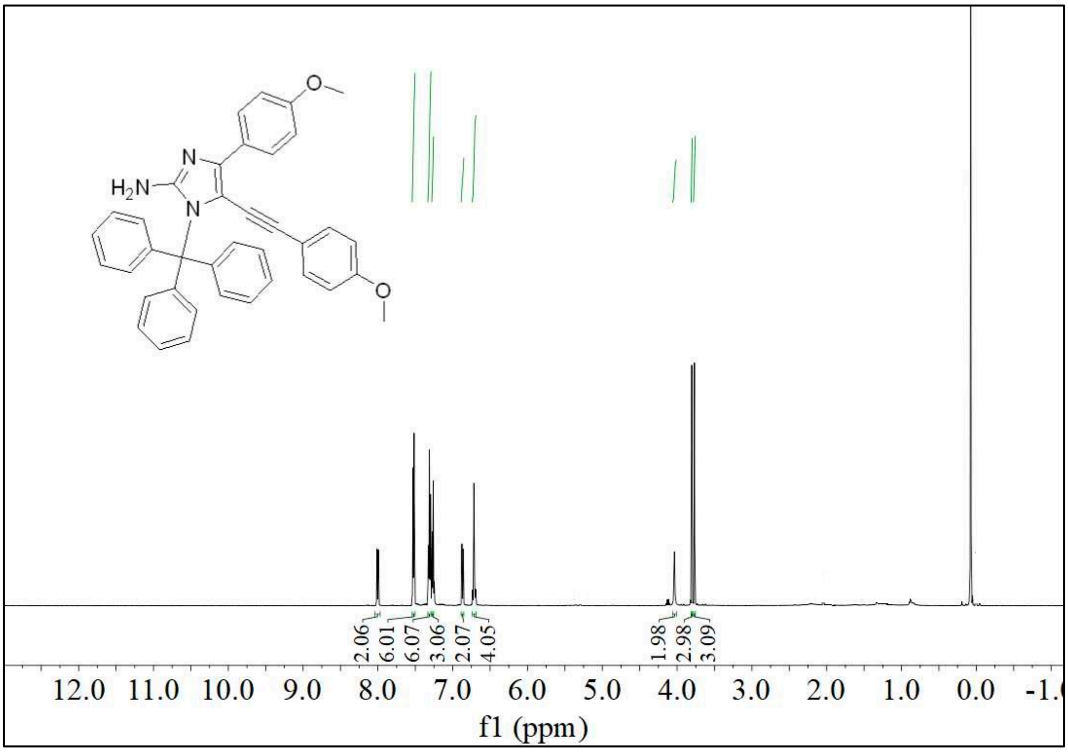

Figure S11 ^{1}H NMR Spectrum of **7** in Chloroform-d

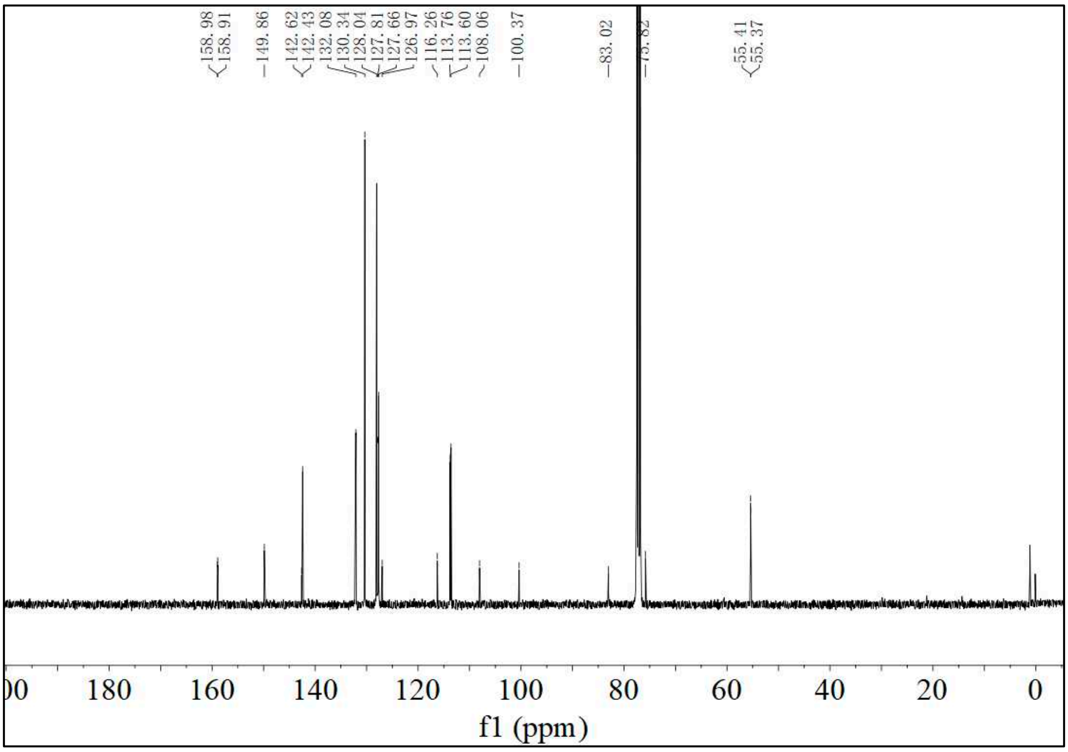

Figure S12 ^{13}C NMR Spectrum of **7** in Chloroform-d

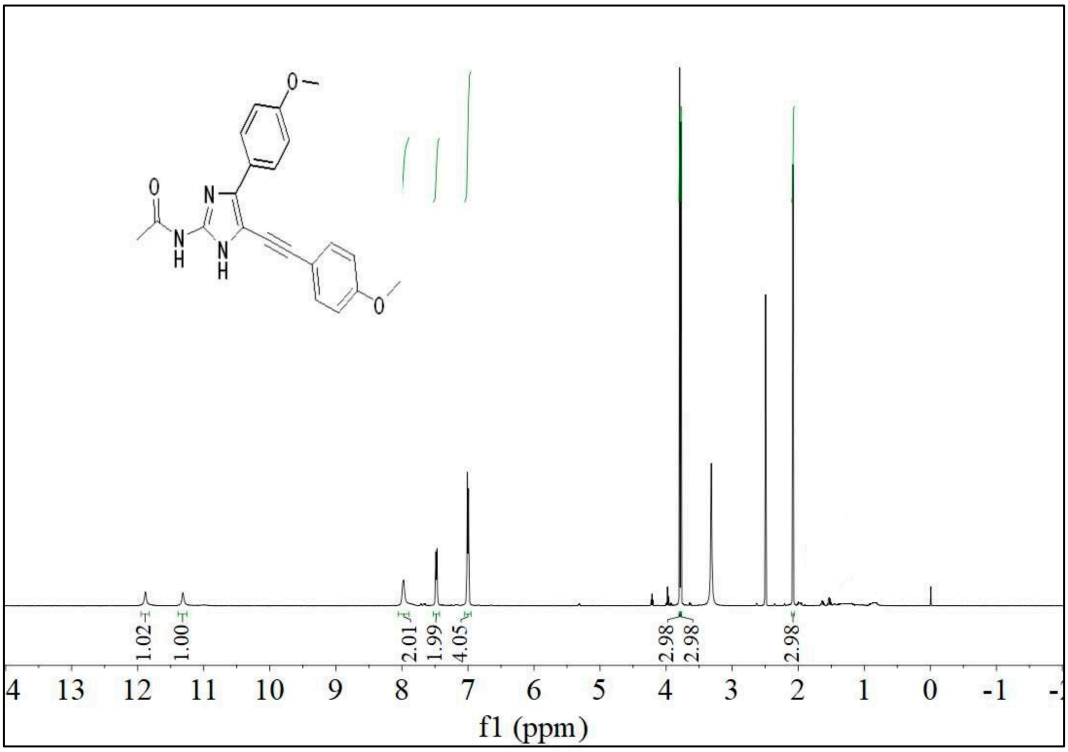

Figure S13 ^{1}H NMR Spectrum of **8** in DMSO-d$_6$

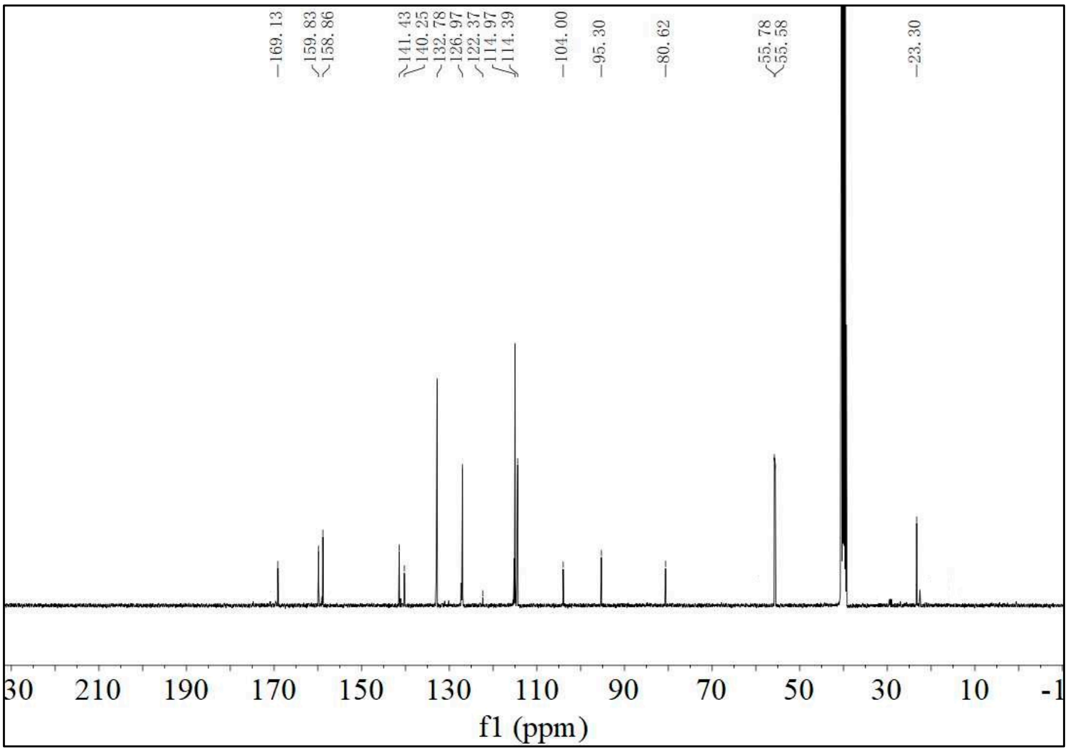

Figure S14 ^{13}C NMR Spectrum of **8** in DMSO-d$_6$

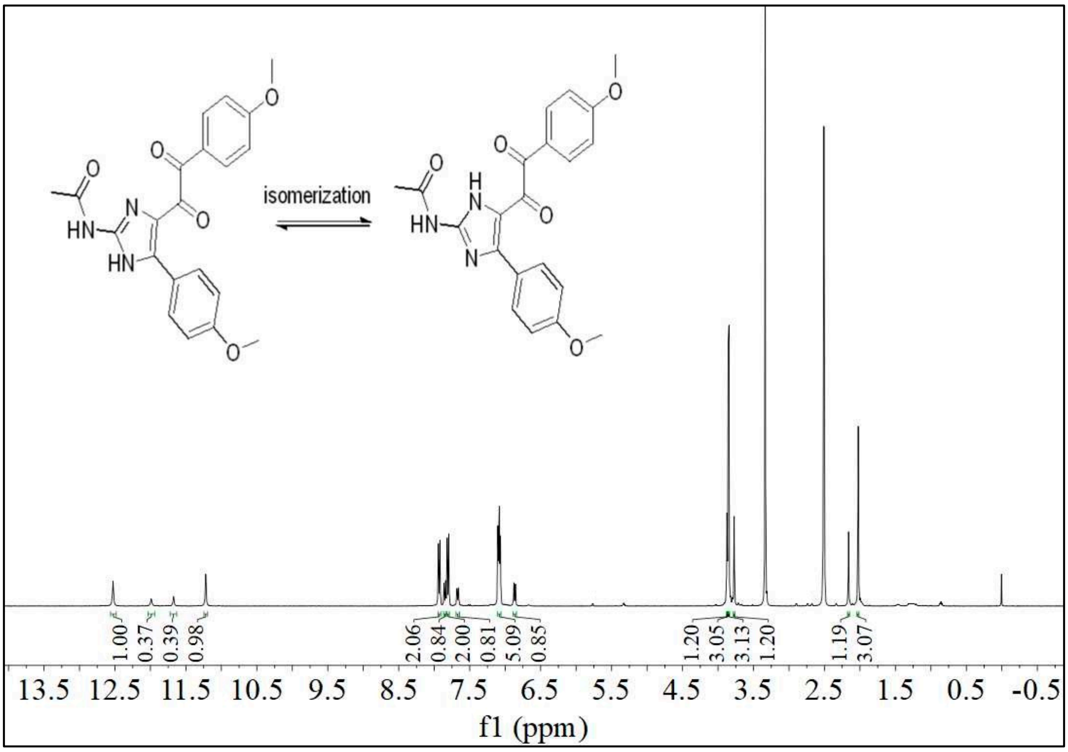

Figure S15 ^{1}H NMR Spectrum of **9** in DMSO-d_6

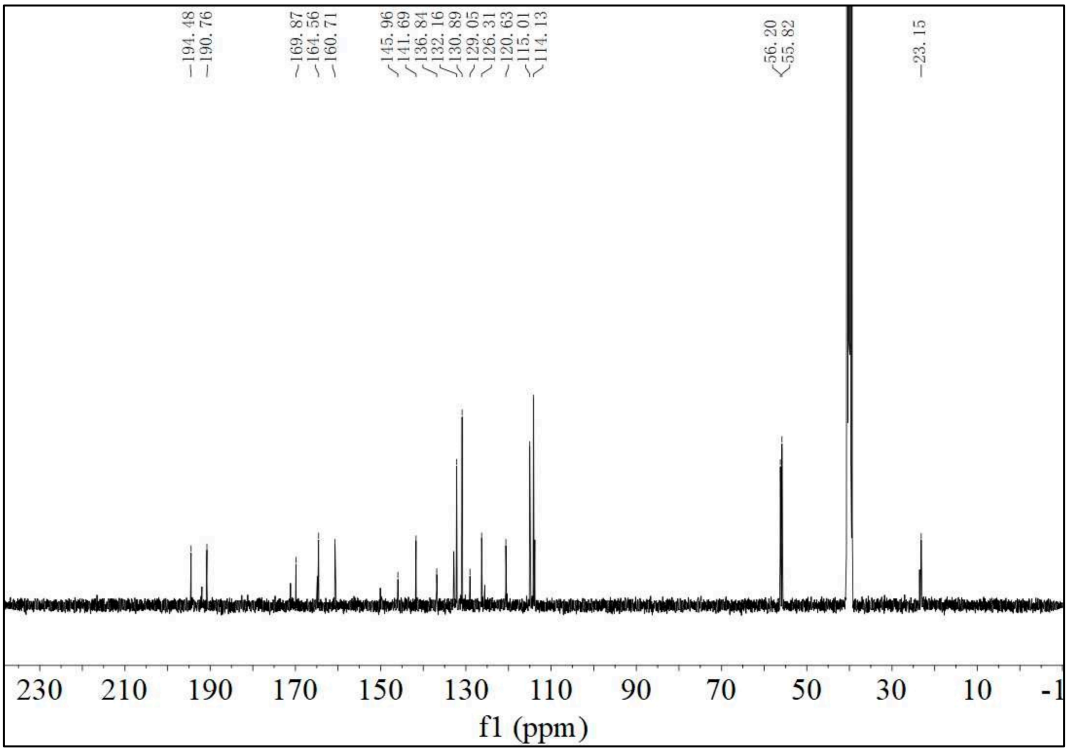

Figure S16 ^{13}C NMR Spectrum of **9** in DMSO-d_6

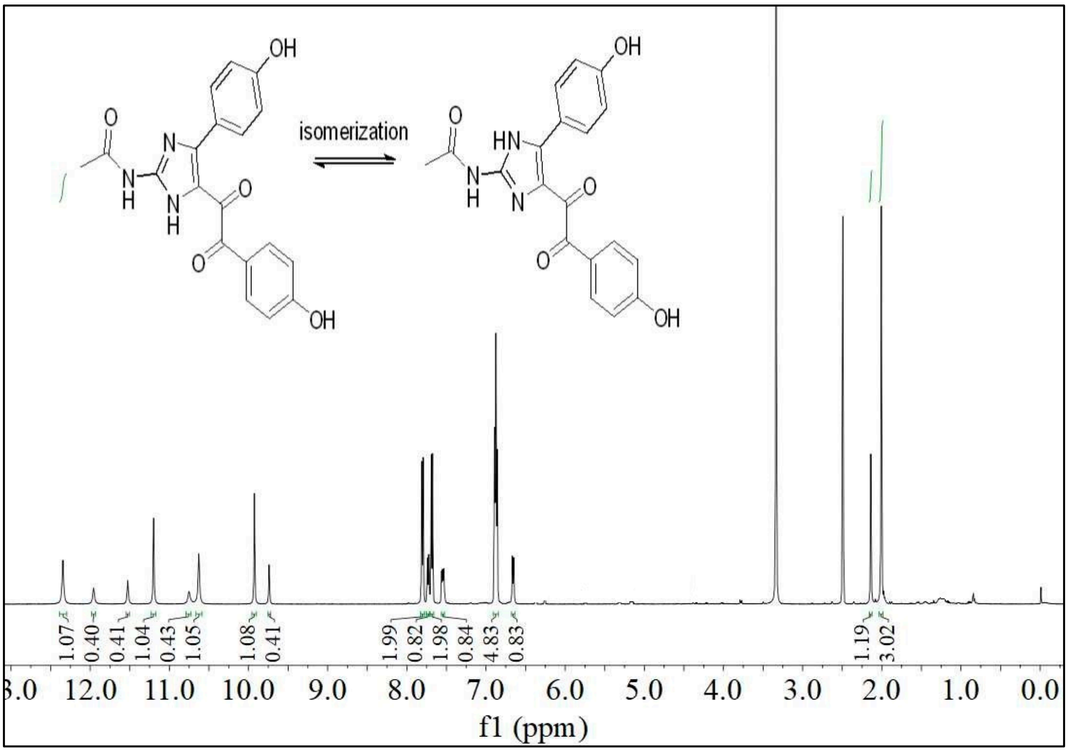

Figure S17 ^{1}H NMR Spectrum of **10** in DMSO-d6

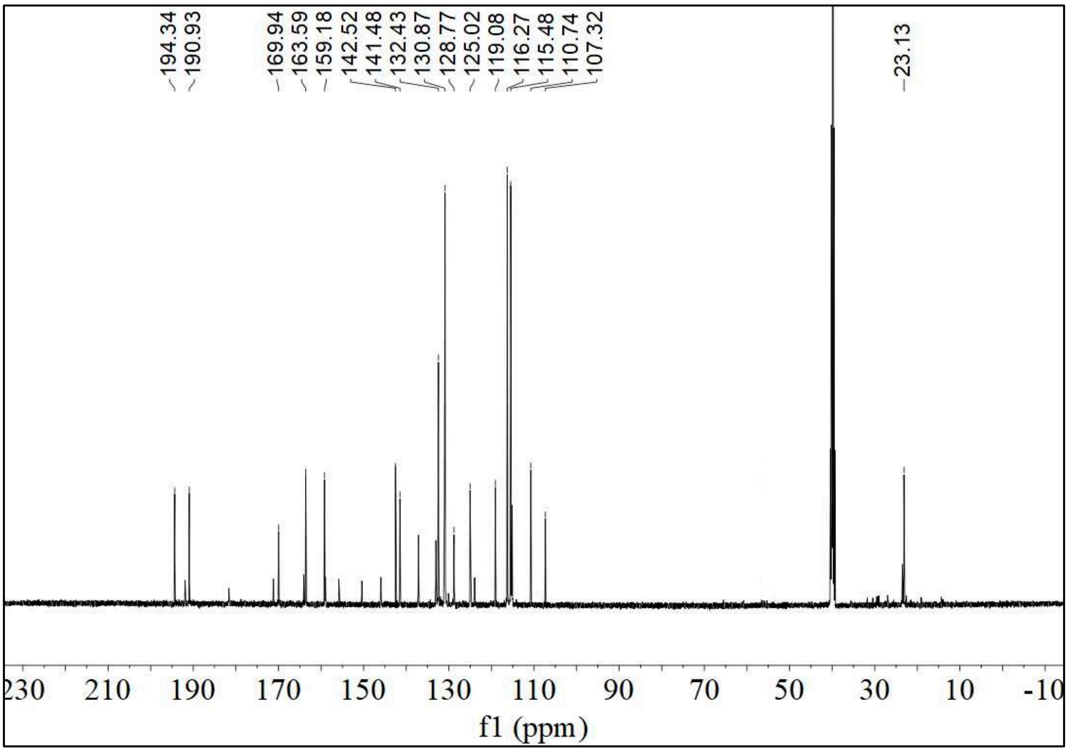

Figure S18 ^{13}C NMR Spectrum of **10** in DMSO-d6

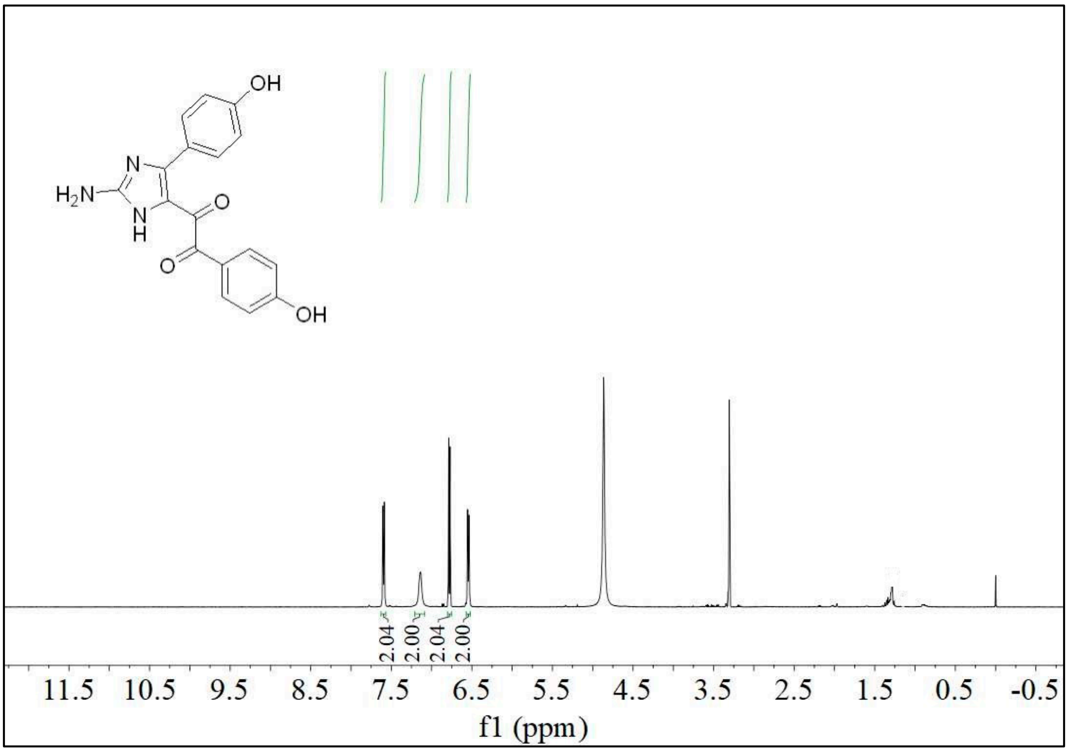

Figure S19 ^{1}H NMR Spectrum of **11** in Methanol-d_4

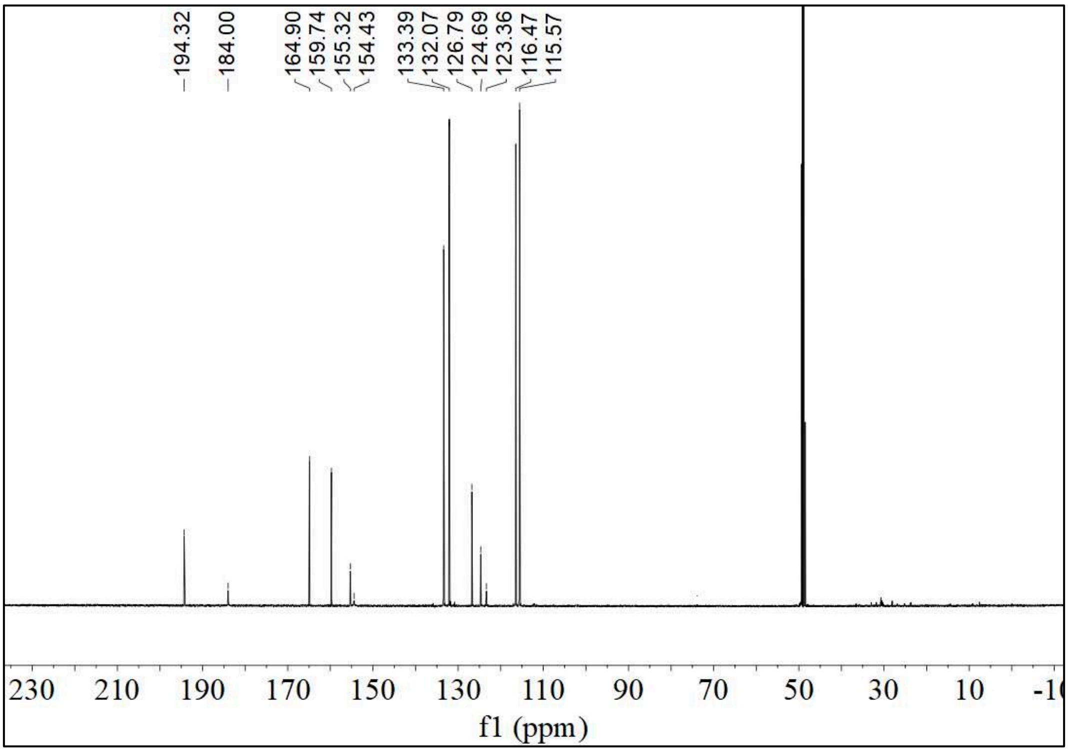

Figure S20 ^{13}C NMR Spectrum of **11** in Methanol-d_4
